# Elevated Aminotransaminases As the First Manifestation of Sarcoidosis

**DOI:** 10.1155/2009/193785

**Published:** 2009-05-26

**Authors:** Georges Nawfal, Christelle Budin, Raymonde Bouvier, Alain Lachaux

**Affiliations:** ^1^Division of Gastroenterolgy, Hepatology and Nutrition, Department of Pediatrics, Hospices Civils de Lyon, University of Lyon 1, F-69003 Lyon, France; ^2^Anatomic Pathology Department, Femme-Mere-Enfant Hospital, Bron, Inserm, U851, FR-128, 69365 Lyon, France

## Abstract

Sarcoidose is a rare disease in children. The aminotransaminase level is often normal to moderately elevated (2 to 3 folds of the normal level). We report the case of a child who presented an aminotransaminase level that was 10 times the normal level, as the first manifestation of sarcoidosis.

## 1. Introduction

Sarcoidosis is a chronic multisystem, granulomatous disorder of unknown etiology [1]. It occurs mainly in adults, with estimated prevalences ranging from <1 to 40 cases per 100 000 population [2]. Milman et al. recently reported that the approximate calculated incidence of sarcoidosis in children in Denmark was 0.27 per 100 000 children per year [3].

In cases of hepatic sarcoidosis, aminotransaminase levels are normal to moderately elevated (2- to 3-folds of the normal level) [4, 5]. Usually this abnormality is not the first manifestation of sarcoidosis. 

We report the case of a child who presented with an aminotransaminase level that was 10 times the normal level, as the first manifestation of sarcoidosis.

## 2. Case Report

A 13-year-old boy was referred to our institution for evaluation of elevated aminotransaminases and *γ*-glutamyl transpeptidase. He presented with weight loss (10 Kg in one year) and anorexia. There was no evidence of pulmonal or neurological symptoms. There was no evidence of any preceding medication. The father and the mother of the patient have a hepatitis B.

The consistence of liver was normal, and it was palpable below the costal margin (+3 cm). Physical examination was otherwise unremarkable.

Laboratory testing showed serum aspartate transaminase of 127 U/L, serum alanine transaminase of 496 U/L, *γ*-glutamyl transpeptidase of 121 U/L, total serum bilirubin of 7 *μ*mol/L, serum albumin of 33.53 g/L (normal 37–50), serum LDH of 227 U/L (normal 135–345), and a normal serum INR. No evidence of viral infections including hepatitis A virus, hepatitis B virus, hepatitis C virus, Epstein-Barr virus, or Cytomegalovirus was found. Antinuclear antibodies (ANAs), anti-Smooth muscle antibodies (ASMAs), and liver-kidney microsomal type 1 (LKM1) antibodies were negative. Ceruloplasmin serum level was 0.348 g/L (normal 0.220–0.610). Ultrasonography showed homogenous hepatomegaly, perihepatic, paraaortic, and mesenteric enlarged lymph nodes (nodes sizes were 10 to 20 millimeters).

**Figure 1:**
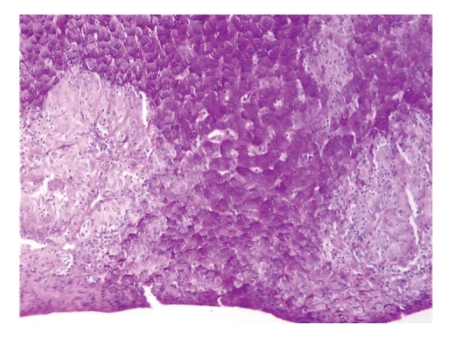
Nonnecrotizing granulomas of hepatic sarcoidosis.

A liver biopsy (Figure 1) revealed aggregated epithelioid histiocytes and multinucleated giant cells with no evidence of necrosis. These lesions were consisting with nonnecrotizing granulomas. There was no evidence of iron overload in the liver biopsy. Tuberculin skin test was negative. Angiotensin-converting enzyme level (ACE) was 139 U/L (normal 8–52 U/L), ESR was 73, and C-reactive protein was 6 mg/L. Thoracic CT-Scan showed numerous enlarged mediastinal lymph nodes. Ophthalmologic exam was normal.

Systemic sarcoidosis with liver involvement was diagnosed, and therapy with corticosteroids 2 mg/kg/d per os was started for 2 weeks. The liver function tests (LFTs) normalized within 1 week of treatment, and ACE level normalized within 2 weeks of treatment. The corticosteroids therapy was decreased slowly to 0.1 mg/kg/d within 2 months but then increased to 1 mg/kg/d because the LFTs and ACE levels increased again. One year later, the patient developed renal interstitial fibrosis and tubular atrophy.

## 3. Discussion

Many diagnoses must be considered in case of hepatic cytolysis like [5] acute and chronic hepatitis (viral, drugs, toxicity, autoimmune), acute obstruction of extrahepatic biliary tract (such as cholelithiasis), primary and secondary malignancy (such as lymphoma), benign tumors (hepatic adenoma, hydatic cyst, etc.), metabolic diseases (Wilson disease, hemochromatosis), *α*1-antitrypsin deficiency, hepatic granulomatosis (sarcoidosis, tuberculosis, brucellosis, etc.), systemic diseases (lupus erythematosus, juvenile dermatomyositis, juvenile rheumatoid arthritis), and celiac disease.

In our patient case, elevated aminotransaminases was associated with weight loss and anorexia. In this case we must first consider [6] primary and secondary malignancies (such as lymphoma). Other diagnoses to consider are [6] systemic pathology (lupus erythematosus, juvenile dermatomyositis, juvenile rheumatoid arthritis, sarcoidosis, autoimmune hepatitis), celiac disease, and infectious diseases (tuberculosis, brucellosis).The *γ*-glutamyl transpeptidase of 121 U/L (3-folds the normal level) did not help us reduce the differential diagnosis. 

Abnormalities of liver function tests (LFTs) in patients with sarcoidosis include [4]: moderately elevated aminotransaminases (2- to 3-folds of the normal level) are found in 20% of patients, moderate anicteric cholestasis (elevated *γ*-glutamyl transpeptidase and/or alkaline phosphatase) is found in 50% to 75% of patients, and rarely, conjugated bilirubin is found elevated. In our case, the patient presented with an aminotransaminase level that was 10 times the normal level.

Ultrasonography is valuable in investigating cytolysis. Our patient had homogenous hepatomegaly, enlarged perihepatic and mesenteric lymph nodes (node sizes were 10 to 20 millimeters). 

The presence of abdominal lymphadenopathies led us retain the following diagnoses: lymphoma, sarcoidosis, infectious processes (tuberculosis, brucellosis), and celiac disease.

Abdominal lymphadenopathies are frequently observed in sarcoidosis. In a study of 11 patients with abdominal or pelvic lymphadenopathies secondary to sarcoidosis, enlarged celiac artery nodes were seen in 82% of the cases, porta hepatis nodes in 73%, paraaortic or paracaval nodes in 73%, gastrohepatic ligament nodes in 55%, mesenteric nodes in 55%, superior mesenteric artery nodes in 45%, and pelvic nodes in 33%. Retrocrural nodes were involved in only 18% of the patients [7]. When comparing patients with sarcoidosis with a group of patients with non-Hodgkin's lymphoma, retrocrural nodes were involved more frequently (70%) in the patients with lymphoma [8]. Mean node size was also significantly smaller in patients with sarcoidosis than in patients with lymphoma (mean ± SD, 2.6 ± 1.7 cm in sarcoidosis versus 8.0 ± 5.5 cm in lymphoma), but diameters of some nodes in patients with sarcoidosis were as high as 7.5 cm [8]. In tuberculosis patients, abdominal lymphadenopathy predilection is for periportal, peripancreatic, and mesenteric locations [9]. Lymphadenopathy is not a common presenting feature in patients with celiac disease but was found in 12% of patients in large series [10].

The size of lymphadenopathies in our patient was in favor of sarcoidosis more than lymphoma.

Liver biopsy is an important step to assess the etiology of transaminitis. In our patient, the liver biopsy revealed numerous non-necrotizing granulomas. Hepatic granulomas have many causes (Table 1) [11]. These involve drugs and systemic disorders (usually infections) more often than primary liver disorders. The infections are important to be recognized because they require specific treatments. Tuberculosis and schistosomiasis are the most important infectious culprits worldwide; viral pathogens are less common. Sarcoidosis is the most important noninfectious cause; the liver is involved in about two thirds of patients and occasionally is the dominant clinical manifestation. Granulomas are much less common in primary liver diseases, of which primary biliary cirrhosis is the only important etiology. Small granulomas occasionally occur in other liver diseases but are of no clinical significance. Idiopathic granulomatous hepatitis is a rare syndrome of hepatic granulomas, recurrent fever, myalgias, fatigue, and other systemic symptoms, often occurring intermittently for years. Some experts believe that it is a variant of sarcoidosis [11].

Angiotensin-converting enzyme (ACE) level was 139 U/L (normal 8–52 U/L). Serum ACE is thought to be produced by the epithelioid cells within sarcoid granulomas. It is elevated in approximately 60% of patients with sarcoidosis, and its level is thought to reflect whole-body granuloma mass and disease activity [12, 13].

Etiology for sarcoidosis remains obscure but probably involves environmental and host factors. Therefore, the diagnosis of sarcoidosis is currently based on a combination of suggestive clinical features with histologically documented noncaseating granulomas, in the absence of other known causes of granuloma formation [14].

In our patient, the presence of elevated aminotransaminases, weight loss, anorexia, enlarged perihepatic paraaortic and mesenteric lymph nodes on ultrasonography, nonnecrotizing granulomas on liver biopsy, high level of ACE, and negative tuberculin skin test was in favor of sarcoidosis.

The treatment of hepatic sarcoidosis depends on clinical and laboratory disease manifestations. When histological appearance of noncaseating granulomas is present without clinical or biochemical liver disease or dysfunction, treatment is not required. When LFTs are abnormal without any evidence of clinical or laboratory systemic sarcoid involvement, treatment remains a controversial issue. This is because untreated patients might demonstrate “spontaneous” LFTs improvement [15].

In conclusion, in a pediatric population, the presence of elevated aminotransaminases (10-folds the normal level) associated with perihepatic and mesenteric enlarged lymph nodes must evoke the diagnosis of sarcoidosis.

## Figures and Tables

**Table 1:** [11].

Causes of hepatic granulomas
*Drugs*
Allopurinol
Phenylbutazone, quinidine
Sulfonamides

*Infections*
*Bacterial* (actinomycosis, brucellosis, cat-scratch fever, syphilis, TB* and other mycobacteria, tularemia)
*Fungal *(blastomycosis, cryptococcosis, histoplasmosis)
*Parasitic* (schistosomiasis*, toxoplasmosis, visceral larva migrans)
*Viral* (cytomegalovirus, infectious mononucleosis, Q fever)

*Liver disorders*
Primary biliary cirrhosis

*Systemic disorders*
Hodgkin's lymphoma, polymyalgia rheumatic, and other connective tissue disorders, sarcoidosis*

*Most common causes
